# Individual and community level factors associated with unintended pregnancy among pregnant women in Ethiopia

**DOI:** 10.1038/s41598-021-92157-4

**Published:** 2021-06-16

**Authors:** Liknaw Bewket Zeleke, Addisu Alehegn Alemu, Eskeziaw Abebe Kassahun, Bewket Yeserah Aynalem, Hamid Yimam Hassen, Getachew Mullu Kassa

**Affiliations:** 1 College of Health Sciences, Debre Markos University, Debre Markos, Ethiopia; 2 University of Antwerp, Antwerp, Belgium

**Keywords:** Health care, Medical research, Risk factors

## Abstract

Unintended pregnancy is among the major challenges of public health and a major reproductive health issue, due to its implications on the health, economic and social life of a woman and her family mainly in low and middle-income countries, particularly sub-Saharan Africa. The study aimed to assess unintended pregnancy and associated factors among pregnant women in Ethiopia using multilevel analysis from the EDHS 2016. We used the data from the 2016 Ethiopian Demographic and Health Survey, comprised of 1122 pregnant women. The prevalence of unintended pregnancy was determined through descriptive statistics and multilevel logistic regression was performed to identify factors associated with unintended pregnancy. Variables with a p-value < 0.05 in the selected model were considered as significantly associated and an adjusted odds ratio was used to determine the strength and direction of the association. The prevalence of unintended pregnancy was 29.7% (CI 27.0%, 32.4%), of which 20.4% were mistimed and 9.3% unwanted. Being multi-para and fertility preference to have no more child were associated with a higher risk of unintended pregnancy whereas husbands' polygamy relation, having no women autonomy, and living in Afar and Somali regions showed a less likely risk of experiencing an unintended pregnancy. This study showed that the proportion of women who experienced unintended pregnancy is considerably high. Parity, fertility preference, polygamy relation, women autonomy, and region were identified factors associated with unintended pregnancy. Therefore, policymakers at all levels, reproductive health experts, and concerned organizations should emphasize minimizing unintended pregnancy targeting the regional variation at large. Researchers have to explore the regional variations through a qualitative study.

## Introduction

Unintended pregnancy is a type of pregnancy either mistimed, occur earlier than the desired time, or totally unwanted, occur when the individuals desire no more children^1,2^. It results from either non-use of any contraceptive or failure of the methods due to incorrect use. Experiencing unintended pregnancy is among the most critical challenges in the public health aspect and it imposes considerable implications on the health, economic and social life of the pregnant woman and her family^3,4^.

It is estimated that 208 million pregnancies occur every year across the globe, of which 41% are unintended^5^. In 2012, 133 pregnancies occurred per 1000 reproductive age women and the rate in developing countries is far higher than in developed countries (140 versus 94). By 2012, the rate of unintended pregnancy was 53 per 1000 worldwide, and Africa accounted for the highest rate (80 per 1000) whereas in the sub-regional perspective eastern and middle Africa took the highest-burden^6^. In sub-Saharan African countries, the prevalence of unintended pregnancy was 29.0% which ranges between 10.8% in Nigeria and 54.5% in Namibia^7^.

Despite the extensive coverage of family planning in Ethiopia and the efforts in this issue, unplanned pregnancies remained a major public health problem. The national prevalence of unintended pregnancy was found to be 24.0% ranging from 1.5% in the Afar region to 39.0% in the Oromia region^8^.

Unintended pregnancy imposes a wide range of physical and psychological health problems, and economic impacts on women, men, family, and society, the global level at large^9–12^.

Various global and local studies identified that maternal age, marital status, education, occupation, partner-related characteristics (age, education, and occupation), residence, number of children, family size, income, wealth status, and obstetric characteristics were found to be associated factors of unintended pregnancy^8,13–24^. In Ethiopia, several studies assessed the prevalence of unintended pregnancy and associated factors in different perspectives and levels like in the national level or local levels but none of them tried to address the determinants of unintended pregnancy in the individuals and community level using multilevel analysis. It is essential to identify factors associated with unintended to design targeted intervention. Hence, this study aimed to identify individual and community-level factors associated with unintended pregnancy among pregnant women in Ethiopia using data from the 2016 Ethiopian Demographic and Health Survey (EDHS).

## Methods and data

### Data source and population

We used data from the nationally representative (9 regions and 2 city administrations) cross-sectional study EDHS 2016^25^. The sampling procedure of the EDHS consists of a two-stage stratified sampling procedure. In the first instance, the regions were stratified in urban and rural areas, and each stratum was clustered into enumeration areas based on the 2007 Ethiopian housing and population census^26^. The first sampling involved the selection of clusters through probability proportional size allocation. Finally, households were selected from each cluster using the systematic probability sampling method.

**Figure 1:**
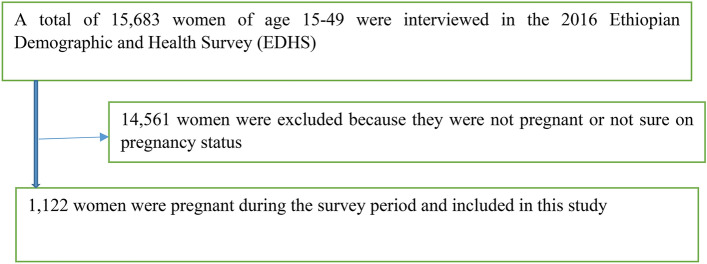
Schematic presentation of selecting the sample from EDHS 2016 data.

For the current analysis, 1122 pregnant women were selected from the total of 15,683 reproductive age women interviewed in the 2016 EDHS (Fig. 1).

### Study variables and definitions

The outcome variable of this study is unintended pregnancy which represents mistimed (the pregnancy that occurs earlier than desired time) or unwanted (the pregnancy that occurs when no more children are desired) types of pregnancy^1^. The EDHS assessed it through a question inquiring from women whether the most recent pregnancy is wanted or not and those who didn’t want the pregnancy were further requested whether they wanted later or not wanted^25^.

#### Individual-level factors

Age, marital status, education, husband education, residence, religion, awareness on modern contraceptive methods, working status, parity, ever terminated pregnancy, fertility preference, sex of household head, household wealth index, autonomy, and polygyny relationship^13,17–22,27–31^.

#### Community-level factors

Residence, region, community women education, community poverty status, community media exposure, community women autonomy, and community modern contraceptive methods awareness^32,33^. Residence and region were taken directly from EDHS, however, other community-level variables namely community women education, community poverty status, community media exposure, community women autonomy, and community modern contraceptive methods awareness were created from individual-level factors by aggregating them using the median due to the asymmetric distribution of the data. In aggregating the community level variables, initially respective individual-level variables were dichotomized into yes or no by referring to previous studies and those clusters with a median of 0.5 and 1.0 were considered as clusters having high proportion, and those with the median value of 0.0 were taken as having low proportion^34^.

#### Community-women education

The proportion of women who completed primary and above educational level in the clusters^35^.

#### Community poverty status

The proportion of women who have poorer and poor wealth status in the clusters^35^.

#### Community media exposure

The proportion of women who had exposure at least for television or radio or newspapers in the clusters^35,36^.

#### Community women autonomy

The proportion of women who make decisions alone or jointly on the woman’s own health care, major household purchases, and visits to the woman’s family or relatives in the clusters^37^.

#### Community awareness on modern contraceptive methods

Refers to the proportion of women in the clusters who have ever heard about modern contraceptive methods.

### Data processing and analyses

The data were accessed in SPSS format from the Demographic Health Survey Archive, and managed and analyzed using R version 3.6.1 (variable recoding and the descriptive statistics) and STATA version 14 software. Due to variation in sample representation, sample weight was considered and the weight variable was created by dividing the individual women sample weight variable by 1,000,000 to make the data representative enough. Then all the descriptive and analytical analyses took this weight into account.

Descriptive statistics were computed to characterize the study population in terms of relevant socio-demographic and obstetric characteristics both at the individual and cluster levels.

We chose relevant factors associated with unintended pregnancy through reviewing previous studies and discussion with experts in the subject matter. We conducted a bivariate analysis using cross-tabulation, to select variables eligible for multivariable analysis. Due to the nature of the EDHS data, being hierarchical, we used multilevel logistic regression to identify variables independently associated with unintended pregnancy using a series of four models.

#### Model I (empty model)

In this model unintended pregnancy was analyzed by the cluster variable to test the random effect of between-cluster variability. The intra-class correlation (ICC) was estimated to determine the effect variability justified by the cluster.

#### Model II

This model was run between the dependent variable and individual-level independent variables.

#### Model III

This model was used to examine the association of community or cluster level variables with unintended pregnancy.

#### Model IV (combined model)

Finally, both individual and community-level variables were run together to examine the combined effect on unintended pregnancy.

The intra-class correlation was calculated for each of the models and the Proportional Change in Variance (PCV) was computed for Model II, III, and IV with respect to the variance in the empty model to show the power of the factors in the model to explain unintended pregnancy by using the formula PCV = (Ve − Vmi)/Ve where Ve is variance obtained in the empty model and Vmi is variance in successive models. PCV was calculated by subtracting the variance of each model from the null model. AIC and BIC were also computed for each of the models and the model with the lowest AIC value (the combined model) was selected to identify individual and community-level factors associated with unintended pregnancy^38^. The fixed-effect sizes of individual and community-level factors on unintended pregnancy were expressed using the Odds Ratio (OR) and the community effect sizes were estimated using the 95% Confidence Interval (95% CI).

### Ethics approval

The data were accessed from Demographic Health Survey Archive through a formal request and permission to analyze the data and dissemination of the results has been obtained.

## Results

### Individual-level characteristics

The median age of participants was 26.0 with a range of 15–48 years. Nearly half (49%) of the respondents were under the age group 25–34 and almost all (97.1%) were married. Slightly higher than half (53.3%) of them and 43.9% of the respondents’ husbands had no formal schooling. Nearly one-fifth (20.2%) were primigravida and 9.7% had terminated pregnancy at least once. One-tenth (9.8%) were in a polygyny relationship. (Table 1).

**Table 1:** Individual-level characteristics of pregnant women from EDHS 2016.

Variable	Frequency (weighted)	Percent (weighted)
**Age**		
15–19	101	8.9
20–24	299	26.3
25–34	556	49.0
35 and above	179	15.8
**Marital status**		
Unmarried	1102	2.9
Married	33	97.1
**Religion**		
Orthodox	317	53.3
Muslim	203	34
Protestant	25	4.2
Catholic and others	50	8.4
**Educational status**		
No formal education	604	53.2
Primary education	398	35.0
Secondary education	94	8.3
College or university	40	3.5
**Working status**		
Yes	839	73.9
No	296	26.1
**Husband educational status**		
No formal schooling	482	43.9
Primary education	411	37.5
Secondary education	129	11.8
College or university	70	6.4
Don’t know	5	0.4
**Sex of HH head**		
Male	985	86.7
Female	151	13.3
**Wealth index**		
Poorest	257	22.7
Poorer	265	23.3
Middle	213	18.8
Richer	198	17.4
Richest	203	17.8
**Awareness on MCM**		
Yes	1107	97.5
No	29	2.5
**Parity**		
Nullipara^a^	229	20.2
Multipara^b^	500	44.0
Grand-multipara^c^	406	35.9
**Ever terminated pregnancy**		
No	1025	90.3
Yes	110	9.7
**Fertility preference**		
Have another	703	61.9
Undecided	71	6.2
No more	362	31.9
**Polygamy relation**		
Yes	108	9.8
No	989	90.2

^a^No history of childbirth.

^b^Mothers who have given birth to 1–4 child.

^c^Mothers who have given birth of five and above child.

### Community-level variables

Above two-fifths, (41.9%) of the respondents were from Oromia region and 85.8% were rural residents. Nearly half of the respondents were from clusters with higher women's education (52.7%) and high poverty (53%) (Table 2).

**Table 2:** Community-level characteristics of pregnant women in EDHS 2016.

Variable	Frequency (weighted)	Percent (weighted)
**Region**		
Tigray	56	4.9
Afar	12	1.1
Amhara	220	19.4
Oromia	475	41.9
Somali	59	5.2
Benishangul	12	1.1
SNNPR	264	23.3
Gambela	3	0.1
Harari	4	0.1
Addis Adaba	24	2.2
Dire Dawa	5	0.1
**Residence**		
Urban	161	14.2
Rural	974	85.8
**Community women education**		
Higher women education	598	52.7
Low women education	537	47.3
**Community poverty**		
High poverty	601	53
Low poverty	534	47
**Community women autonomy**		
High	913	80.9
low	214	19.1
**Community media exposure**		
High	456	40.2
Low	679	59.8
**Awareness on MCM**		
High	1123	98.9
Low	12	1.1

### Prevalence of unintended pregnancy

**Figure 2:**
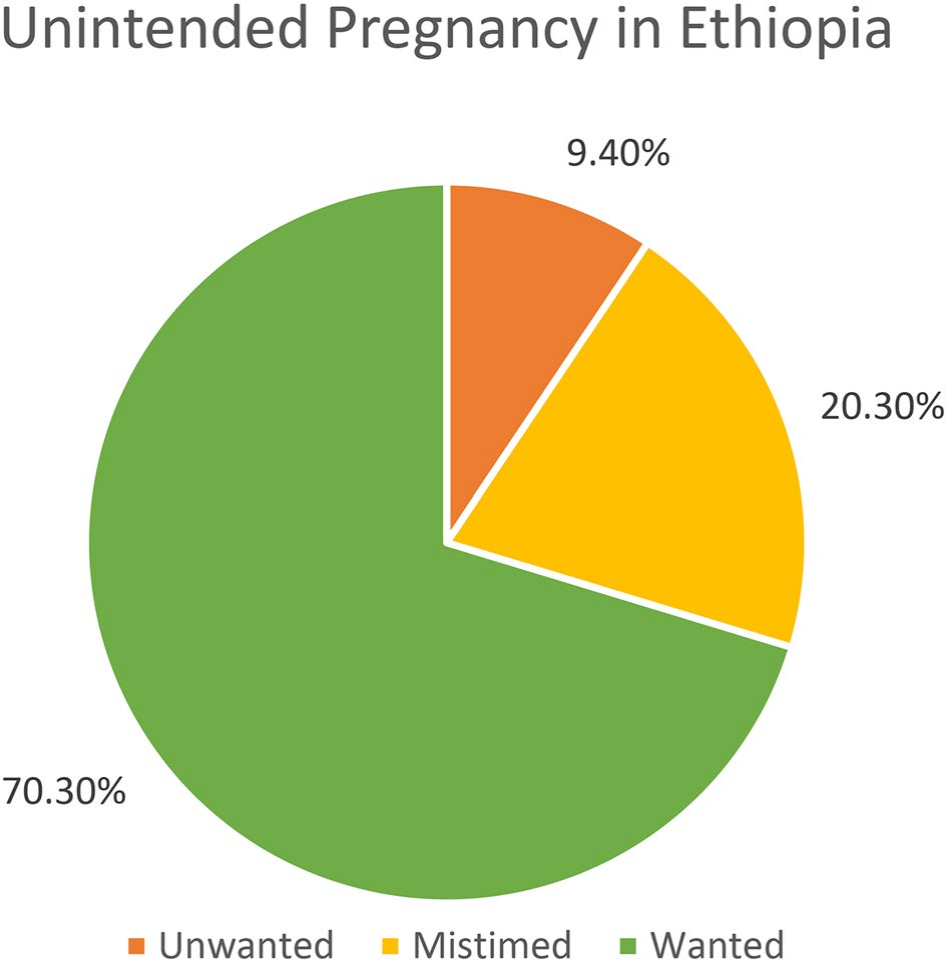
The prevalence of unintended pregnancy among pregnant women in Ethiopia, 2016.

Three hundred thirty-three (29.7%, CI 27.0%, 32.4%) pregnancies were unintended, comprised of 20.3% mistimed and 9.4% unwanted (Fig. 2).

### Factors associated with unintended pregnancy

In the multilevel logistic regression four models had been fitted. The random effect analysis in the null model was used to examine the cluster effect on unintended pregnancy. The result depicted a significant variability in unintended pregnancy across the clusters (ICC 0.193) which further indicates that the cluster accounted for a 19.3% variance in unintended pregnancy. The ICC values for models II, III, and IV respectively are 0.114, 0.005, and 0.043.

The fixed effect analysis indicated that parity, fertility preference, polygamy relation, and women autonomy from individual-level factors, and region from community-level factors were found to be significantly associated with unintended pregnancy (Table 3). Women who were para 1–4 were 3.49 [AOR 3.49, CI (1.71, 7.15)] and grand multipara were 4.86 [AOR 4.86, CI (2.03, 11.61)] times more likely to have unintended pregnancy as compared to nulliparous women. Women who no more wanted children were 2.54 [AOR 2.54, CI (1.68, 3.85)] times more likely to experience unintended pregnancy compared with women who want to have more children. Women whose partners have polygamy relations had 55% [AOR 0.45, CI (0.23, 0.85)] less likely to have unintended pregnancy as compared to their counterparts. Women who had autonomy in decision making in some household tasks were 35% [AOR 0.65, CI (0.45, 0.95)] less likely chance to experience unintended pregnancy as compared to those who had no autonomy. Women who were living in Afar and Somali regions were 81% [AOR 0.19, CI (0.06, 0.58)] and 93% [AOR 0.07, CI (0.02, 0.25)] less likely chance to have unintended pregnancy than women from the Tigray region.

**Table 3:** Factors associated with unintended pregnancy were identified through multilevel logistic regression.

Variable	Null model	Model II	Model III	Model IV
**Age**				
15–19		1.00		1.00
20–24		0.62 (0.28, 1.39)		0.57 (0.25, 1.29)
25–34		0.62 (0.28, 1.41)		0.51 (0.22, 1.17)
35 and above		0.72 (0.28, 1.84)		0.60 (0.23, 1.57)
**Marital status**				
Married		1.00		1.00
Unmarried		2.21 (0.38, 12.96)		2.34 (0.39, 13.85)
**Religion**				
Muslim		1.00		1.00
Orthodox		1.55 (0.99, 2.43)		0.90 (0.51, 1.59)
Protestant		1.86 (1.14, 3.06)*		0.89 (0.50, 1.56)
Catholic and others		2.74 (0.99, 7.57)		1.30 (0.46, 3.72)
**Working**				
No		1.00		1.00
Yes		1.35 (0.91, 2.01)		1.40 (0.97, 2.15)
**Parity**				
Nulliparous		1.00		1.00
Multipara		2.85 (1.41, 5.75)**		3.49 (1.71, 7.15)**
Grand multipara		3.36 (1.43, 7.88)**		4.86 (2.03, 11.61)***
**Fertility preference**				
Have another		1.00		1.00
Undecided		1.69 (0.77, 3.73)		1.81 (0.53, 2.62)
No more		3.63 (2.40, 5.48)***		2.54 (1.68, 3.85)***
**Wealth index**				
Poorest		1.00		1.00
Poorer		1.44 (0.86, 2.40)		0.89 (0.46, 1.36)
Middle		1.02 (0.57, 1.83)		0.55 (0.30, 1.02)
Richer		1.28 (0.72, 2.28)		0.71 (0.39, 1.30)
Richest		0.97 (0.55, 1.69)		0.64 (0.28, 1.47)
**Polygamy relation**				
No		1.00		1.00
Yes		0.41 (0.23, 0.77)**		0.45 (0.23, 0.85)*
**Women autonomy**				
No		1.00		1.00
Yes		0.67 (0.47, 0.97)*		0.65 (0.45, 0.95)*
**Residence**				
Urban			1.00	1.00
Rural			1.85(1.13, 3.02)*	1.05(0.46, 2.41)
**Region**				
Tigray			1.00	1.00
Afar			0.23 (0.10, 0.57)**	0.19 (0.06, 0.58)**
Amhara			1.23 (0.61, 2.45)	1.01 (0.44, 2.29)
Oromia			1.81 (0.97, 3.39)	1.48 (0.64, 3.45)
Somali			0.12 (0.05, 0.32)***	0.07 (0.02, 0.25)**
Benishangul			0.47 (0.20 1.10)	0.36 (0.13, 1.01)
SNNPR			1.77 (0.94, 3.34)	1.57 (0.68, 3.62)
Gambela			1.17 (0.53, 2.59)	0.97 (0.35, 2.69)
Harari			0.78 (0.35, 1.73)	0.62 (0.22, 1.75)
Addis Adaba			1.22 (0.44, 3.43)	0.93 (0.29, 2.99)
Dire Dawa			1.69 (0.75, 3.81)	1.19 (0.42, 3.36)
**Random effect**				
ICC	0.193	0.114	0.005	0.043
PCV	NA	46.2%	97.7%	0.81.3%
**Model fitness**				
Log likelihood	− 556.2372	− 466.1785	− 505.7895	− 435.05964
AIC	1116.474	972.3569	1037.579	932.1193
BIC	1126.52	1072.236	1102.876	1086.931

Signif. codes: ‘***’ < 0.001 ‘**’ < 0.01 ‘*’ < 0.05.

## Discussion

This report analyzed the EDHS 2016 data to determine the prevalence of unintended pregnancy among pregnant mothers and to identify associated factors through multilevel analysis. The prevalence of unintended pregnancy was found to be 29.7% (CI 27.0%, 32.4%) which is consistent with study findings conducted in Wolkaite Woreda (26.0%)^29^, Felege Hiwot Referral Hospital (26.0%)^30^, Addis Zemen Hospital (26.1%)^13^, Michew town (29.7%)^17^, Gelemiso General hospital (27.1%)^18^, Arerti town (29.9%)^31^, Port-Said City, Egypt (31.3%)^39^ and sub-Saharan Africa (29.0%)^7^.

The finding is lower than study findings conducted in Duguna Fango District, Wolaita Zone, Southern Ethiopia (36.6%)^19^, Bale Zone, Oromiya Region, Southeast Ethiopia (37.3%)^20^, in Ganji woreda, west Wollega Oromia region (36.5%)^21^, rural Ghana (70%), Arsi Negele Woreda, West Arsi Zone (41.5%), Jimma town, southwest Ethiopia (36.5%)^27^, Hosanna Town, Southern Ethiopia (34%)^22^, DHS analysis in Kenya (40%)^40^, KwaZulu-Natal, South Africa (64.33%)^41^. However, this finding is higher than study findings undertaken in Tepi General Hospital Sheka Zone (22.3%)^28^, EDHS 2016 report (25%)^25^, Nairobi, Kenya (24%)^42^, Urban sites in Senegal (14.3%)^23^ and Iran (21.2%)^43^. The discrepancy might be attributed to differences in the study population for instance some of the previous studies were conducted among reproductive-age women. It might be also attributed to socioeconomic differences among the study populations.

In the multilevel analysis; parity, fertility preference, polygamy relation, and women autonomy and region showed a significant association with unintended pregnancy. Multiparous and grand multiparous women had a more likely chance of experiencing as compared to nulliparous women. Similar findings were obtained by studies conducted in Ethiopia (the National Survey, Addis Zemen hospital, Maichew Town, Gelemso General Hospital, Duguna Fango District, and Arsi Negele Woreda)^8,13,17–19,24^, Senegal^23^, in rural Ghana^14^, in the Amazon basin of Ecuador^44^ and by A Narrative Review of studies in Developing Countries^16^. The increased likelihood of experiencing unintended pregnancy with increased parity might have resulted from reporting differences that mean primiparous women might accept the pregnancy and report it as wanted whereas multiparous women report it properly.

Women who have no more desire to have children had a more likely chance of experiencing unintended pregnancy. A research finding which was also conducted in Jimma town, southwest Ethiopia^27^ also showed a similar association stating that women who desire less number of children were more likely to have unintended pregnancy. This might be due to the difference in reporting, women who have a desire to have more children may accept the pregnancy and later on consider it as wanted and report it as an intended pregnancy. A significant association between husband fertility desire and unintended pregnancy has been reported by several studies in Ethiopia^19,21,22^.

Women whose husbands were in a polygamy relationship were less likely to experience unintended pregnancy. No previous study established a relation between polygamy relationship and unintended pregnancy. Thus, this finding will help future researchers to determine the relationship through the application of advanced study designs that indicate cause and effect relationships.

Regarding women's autonomy, women who were autonomous in household decision-making had less likelihood of having unintended pregnancies as compared to women who were not autonomous. Similar findings were obtained by numerous studies conducted in Ethiopia^16,20,24^ and Senegal^23^. Women’s autonomy in household decision-making has a positive impact on maternal healthcare service utilization^15,45–48^. Unintended pregnancy can be prevented through the utilization of the right contraceptive method at the right time, which is among the core maternal health care services.

This study also identified that region of residence has a significant association with unintended pregnancy. Women living in Afar and Benshangul Gumuz regions tend to have less likelihood of having unintended pregnancies as compared to women living in the Tigray region. This might be attributed to fertility desire difference because Afar and Benshangul Gumuz are underdeveloped regions whereas Tigray is among developed regions in the Ethiopia context. A similar geographic difference in the prevalence of unintended pregnancy was reported by a multilevel analysis of demographic health survey conducted in the amazon basin of Ecuador^44^.

### Strengths and limitations

As a strength, the study is representative at the national level and it is the analysis of the data from pregnant mothers which helps to reduce recall bias. However, the design of the study is limited to establish cause and effect relationship.

## Conclusion and recommendation

This study revealed that a significant proportion of pregnant women experience unintended pregnancy. Women who were multi-para and multipara, fertility preference to have no more child had a higher risk whereas women whose husbands had polygamy relation, have no autonomy, and Afar and Somali region residents had a less likely risk of experiencing unintended pregnancy. Designers and implementers of projects targeting to avert unintended pregnancy have to consider the regional variations. Various stakeholders have to work in promoting women’s autonomy in the household decision-making role. The authors would like also to recommend for researchers investigate the impact of polygyny relationships on unintended pregnancy using various designs. Researchers should explore the reasons for regional variation in terms of unintended pregnancy prevalence.

## Data Availability

The study used the 2016 Ethiopian Demographic and Health Survey. The data is available and can be obtained from the DHS (https://dhsprogram.com/Data/) upon request.
